# Fatal Neurologic Disease and Abortion in Mare Infected with Lineage 1 West Nile Virus, South Africa

**DOI:** 10.3201/eid1708.101794

**Published:** 2011-08

**Authors:** Marietjie Venter, Stacey Human, Stephanie van Niekerk, June Williams, Charmaine van Eeden, Frank Freeman

**Affiliations:** Author affiliations: University of Pretoria, Pretoria, South Africa (M. Venter, S. Human, C. van Eeden, S. van Niekerk, J. Williams);; National Institute for Communicable Diseases, Sandringham, Johannesburg, South Africa (M. Venter);; Ceres Veterinary Hospital, Western Cape, South Africa (F. Freeman)

**Keywords:** zoonoses, vector-borne infections, West Nile virus, lineage 1, abortion, horses, emerging, viruses, dispatch, South Africa

## Abstract

In 2010, lineage 1 West Nile virus was detected in South Africa in the brain of a pregnant mare that succumbed to neurologic disease and in her aborted fetus, suggesting an association with abortion in horses. All West Nile virus strains previously detected in horses and humans in South Africa were lineage 2.

West Nile virus (WNV), a mosquito-borne flavivirus, may cause outbreaks of febrile disease and encephalitis in humans and horses. Although <1% of human patients experience severe disease (1), up to 90% of symptomatic cases in horses result in neurologic disease with case-fatality rates of 30%–40% (2). In sheep, WNV infection may result in abortion, stillbirth, and neonatal death (3). In humans, transmission by transplacental route and breastfeeding has been described. Congenital WNV infection has been accompanied by bilateral chorioretinitis and severe malformation of the fetal central nervous system (4). We report a case of WNV with fatal neurologic disease and abortion in a horse.

Five genetic lineages of WNV exist, the major 2 being lineages 1 and 2 (5,6). Lineage 1 is distributed widely in North and South America, Europe, parts of Asia, North Africa, and Australia. Lineage 2 strains have been identified in humans and horses with febrile and neurologic disease in southern Africa and Madagascar (7) and recently emerged in central Europe causing encephalitis in birds, humans, and horses (7,8). WNV has become recognized as an important horse pathogen in South Africa with all cases positive by nucleic acid detection or virus isolation belonging to lineage 2 (6,7).

Bird deaths due to WNV are rare in South Africa, probably because of the long-term endemic nature of the virus, which limits their use in sentinel surveillance (9). A positive correlation exists between occurrence of symptomatic equine and human cases, which suggests equine outbreaks might predict disease risk for humans (3).

## The Study

Over the past 3 years, the zoonosis group, Department Medical Virology, University of Pretoria, has investigated horses as sentinels to detect WNV activity in South Africa. The study was approved by the University of Pretoria ethical committee.

Horses are economically important in South Africa, and diagnoses for animals with severe or fatal disease are frequently requested because of the presence of African horse sickness virus in the country, which is a notifiable disease (http://www.nda.agric.za/vetweb). As part of a WNV surveillance program, in an attempt to determine the contribution of WNV to neurologic infections in animals we invited veterinarians throughout the country to report cases of neurologic disease in horses for free diagnosis. Findings of the first year of this study were published in 2009 (10). WNV was identified for up to 21% of undiagnosed neurologic cases in horses. All cases detected by real-time reverse transcription PCR (RT-PCR) belonged to lineage 2, and 70% of cases were fatal or resulted in the horse being euthanized (10).

On May 24, 2010, a 7.5-month pregnant, 8-year-old thoroughbred mare was found recumbent on a farm outside Ceres in the Western Cape. Temperature was within normal limits, mucous membranes indicated mild toxicity, and tongue tone was normal with mild fasciculations. She was treated with intravenous fluids, dimethyl sulfoxide, and cortisone. When rolled over, she got up but had severe hindquarter incoordination and went down again after 1 minute. She was treated with penicillin twice a day, phenylbutazone, and vitamin B1 over the next 3 days. She rose a few times but went down again and was not able to get up on days 4 or 5. She aborted on day 6 and died on day 7. The brain from the fetus was sampled after abortion, and the brain from the mare was examined postmortem; samples were submitted to the WNV surveillance project. Half of the mare's brain was sent in formalin to the University of Pretoria, Onderstepoort, Faculty of Veterinary Sciences for histopathologic examination.

**Figure 1:**
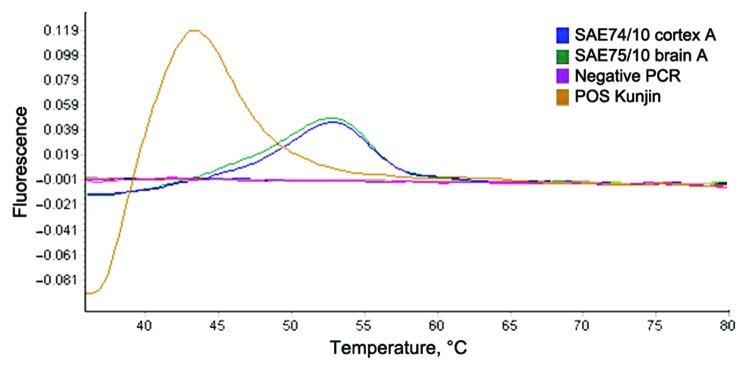
Dissociation curve analysis of the diagnostic nested real-time reverse transcription PCR of West Nile virus isolated from a mare and fetus with fatal neurologic disease, South Africa, 2010. Positive control (Kunjin L1b); SAE74/10 (fetus); SAE75/10 (mare). Expected melting peak of lineage 2 = lineage 1b+6°C; lineage 1a = lineage 1b+10°C.

**Figure 2:**
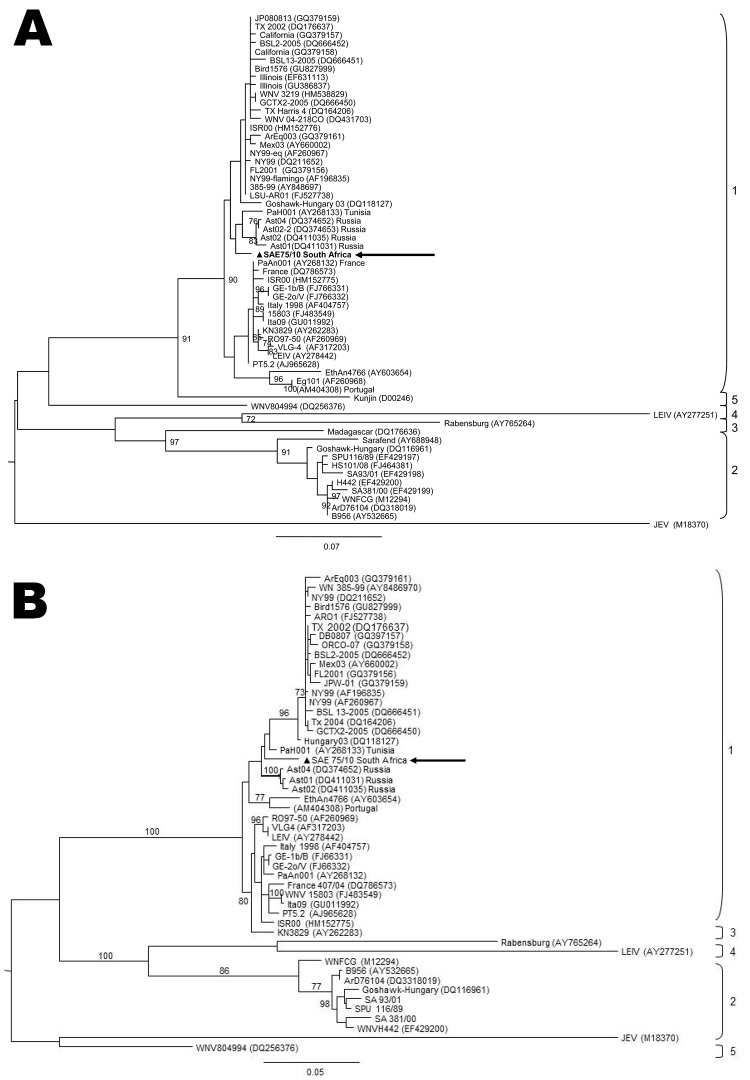
Maximum-likelihood comparison of a partial section of the E-protein gene (A) and NS5 gene (B) of West Nile virus (WNV) lineage 2 strains isolated in South Africa (SA) in 2010 from a mare with fatal neurologic disease and representative sequences of other WNV lineages from various regions of the world. The lineage 1 strain, SAE75/10, identified in South Africa is indicated by a triangle and arrow. Sequences were aligned with MAFFT version 6 (http://align.bmr.kyushu-u.ac.jp/mafft/software) and edited with BioEdit version 7.0.9.0 (www.mbio.ncsu.edu/bioedit). Maximum-likelihood midpoint rooted trees were drawn by using PHYML (www.atgc-montpellier.fr/phyml) under 100 bootstrap repetitions and the HKY codon position substitution model and drawn to scale with the bars indicating 0.07 nt substitutions. Only bootstrap values >70 are shown. Reference strains used from GenBank that were most closely related to SA L1: PAH001, Tunisia (AY268133); Ast04-2-824A, Russia (DQ374652); Ast02-2-25, Russia (DQ374653); Ast02-2-692, Russia (DQ411031); Ast01-187, Russia (DQ411035); Ge1b/B, Spain (FJ766331); GE-2o/V, Spain (FJ766332); WNV Italy-1998-equine, Italy (AF404757); WNV15803, Italy (FJ483549); and Ita09, Italy (GU011992).

Viral RNA was extracted from 30-mg brain sections with the QIAGEN RNeasy Plus Mini Kit (QIAGEN, Valencia, CA, USA). WNV nonstructural protein 5–specific nested real-time RT-PCR that distinguishes lineages 1 and 2 by hybridization probe-melting curve analysis (11) detected WNV lineage 1 in the brains of the mare and foal (Figure 1) and confirmed by sequencing (results not shown, genome positions 9091–9191). A larger region of the NS5 gene (788 bp) of the mare could be amplified by a nested RT-PCR (12) as well as the E-gene (7). Maximum-likelihood analysis clustered these sequences with lineage 1 strains from North Africa and Europe (Figure 2). Strain PAH001 from Tunisia isolated in 1997 from a person who died of neurologic disease (13) was most closely related (p-distance 2.2%; MEGA4, www.megasoftware.net) followed by strains from Russia (p-distance 2.7%–2.9%). Lineage 2 WNV strains were 17.8%–19.4% different from this strain, SAE75/10. E-protein analysis also grouped SAE75/10 with isolates from Russia (Ast02–2-692, Ast02–2-25) and Tunisia (PAH001) with p-distances of 2.3% and 2.7%, respectively. Lineage 2 WNV strains differed by 23.6%–23.9%.

Light microscopic examination of hematoxylin and eosin–stained formalin-fixed wax-embedded cerebellum and cerebrum sections of the mare showed mild to moderate nonsuppurative meningoencephalitis, with olfactory lobe and cortex showing the most pronounced lesions. Lesions included mononuclear perivascular cuffing, glial flares in the cerebellar molecular region, mulitfocal glial nodules, diffuse white matter gliosis, vascular congestion, and macroscopically visible intermittent vascular distention with blood, especially in the thalamus. Occasional Purkinje neurons had nuclear chromatolysis. Unfortunately, brain and other tissues of the aborted foal were not formalin fixed.

Continued surveillance over the past 3 years identified lineage 2 strains as the causative agent in all other acute WNV cases positive by RT-PCR and will be reported in more detail elsewhere. Routine use of real-time nested RT-PCR that distinguish lineage 1 and 2 by hybridization probe analysis (12) resulted in rapid detection of a lineage 1 strain as an emerging pathogen in the country. This technique is especially useful in surveillance studies where routine virus isolation is not attempted due to low viremia and suboptimum specimens from horses such as this.

This strain appears to have been associated with abortion of the fetus and death of the mare. To our knowledge, the only published report identified of natural WNV infection of pregnant mares was of 8 lineage 1 WNV–infected horses with neurologic signs from New York and New Jersey, USA, of which 3 were pregnant but none aborted (14). As in the case we report, 7/8 horses received dimethyl sulfoxide, flunixin meglumine, and phenylbutazone intravenously, and 2 dexamethasone intravenously for 1–3 days. Corticosteroids are regarded as ineffective in the induction of abortion in mares unless large repeated doses are used (15).

## Conclusions

This report of a lineage 1 WNV strain emerging in South Africa confirms the sensitivity of horses as sentinels for detecting new strains and WNV activity in WNV-endemic countries. Continued surveillance will determine if lineage 1 is sustained in this region. In addition, transplacental transmission of WNV in the horse suggests a risk for abortion in pregnant mares with severe neurologic WNV disease.
